# High VSX1 expression promotes the aggressiveness of clear cell renal cell carcinoma by transcriptionally regulating FKBP10

**DOI:** 10.1186/s12967-022-03772-2

**Published:** 2022-12-03

**Authors:** Wenliang Ma, Xin Li, Lei Yang, Jun Pan, Yi Chen, Yanwen Lu, Xiang Dong, Dongmei Li, Weidong Gan

**Affiliations:** 1 Department of Urology, Affiliated Drum Tower Hospital, Medical School of Nanjing University, No. 321 Zhongshan Road, Nanjing, 210008 Jiangsu People’s Republic of China; 2 Immunology and Reproduction Biology Laboratory & State Key Laboratory of Analytical Chemistry for Life Science, Medical School, Nanjing University, Nanjing, 210093 Jiangsu People’s Republic of China; 3 Jiangsu Key Laboratory of Molecular Medicine, Nanjing University, Nanjing, 210093 Jiangsu China

**Keywords:** VSX1, Clear cell renal cell carcinoma, Migration, Invasion, Transcriptional regulation

## Abstract

**Background:**

Clear cell renal cell carcinoma (ccRCC), the most common urological malignancy, has an unfavorable prognosis and an unknown mechanism of progression. Through survival analyses screening of The Cancer Genome Atlas (TCGA) dataset, we identified Visual system homeobox1 (VSX1) as a novel potential prognostic biomarker in ccRCC and subsequently investigated the oncogenic role of VSX1 in ccRCC.

**Methods:**

The differential expression of VSX1 in human tumors and the clinical prognoses were analyzed in the TCGA dataset and Gene Expression Omnibus. Spearman’s correlation coefficient was determined for the correlation analysis of VSX1 expression and other genes of interest. The roles of VSX1 in cell proliferation, invasion, and migration of ccRCC cells were evaluated via the CCK-8 assay, colony formation assay, and Transwell assay, respectively. Further results were demonstrated by western blotting, immunohistochemistry, qRT-PCR, tumor sphere formation, flow cytometry, and the dual‑luciferase reporter assay.

**Results:**

VSX1 mRNA upregulation was generally observed in multiple human malignancies from the TCGA database and was confirmed in ccRCC clinical specimens from our department. High VSX1 expression usually indicated that overall and disease-free survival were unfavorable for patients with ccRCC. In terms of mechanism, knockdown or overexpression of VSX1 affected ccRCC aggressiveness in vitro. The dual-luciferase reporter gene assay implied that VSX1 overexpression significantly increased the luciferase activity of *TMEM44*, *FKBP10*, and *TRIB3*, which indicated that VSX1 promoted ccRCC invasiveness via transcriptional regulation of these genes. The significantly enhanced growth in vitro that was induced by stable VSX1 overexpression was almost restored to normal by the knockdown of *FKBP10*.

**Conclusions:**

This study demonstrated that VSX1 was a novel prognostic biomarker in ccRCC and that high VSX1 expression promoted cell proliferation, invasion, and migration in ccRCC via transcriptional activation of downstream target genes.

**Supplementary Information:**

The online version contains supplementary material available at 10.1186/s12967-022-03772-2.

## Background

Renal cell carcinoma (RCC) is a common urological malignancy with an increasing global incidence, especially among the younger population [1]. From 2013 to 2016, new cancer cases and deaths from RCC in China highlighted the growing burden of this disease on the healthcare system [2, 3]. Clear cell RCC (ccRCC), the most common histological subtype of kidney cancer, accounts for most renal cancer deaths [4, 5]. More than 25% of patients with ccRCC had metastases when initially diagnosed [6]. Although tyrosine kinase receptor inhibitors and immune checkpoint inhibitors have been used to treat metastatic RCC for many years, the development of primary or acquired drug resistance is associated with limited survival benefits in patients [7, 8]. The unfavorable clinical prognoses and limitations of existing treatments mean there is an urgent need to explore the novel molecular mechanisms underlying ccRCC and develop more effective anti-tumor therapies. In the present study, based on survival analyses screening of The Cancer Genome Atlas (TCGA) database, visual system homeobox1 (VSX1) was identified as a novel potential activator gene of ccRCC.

VSX1, also known as CAASDS, is a transcription factor containing the paired-like homeodomain and binds to the locus control region of the visual pigment gene cluster [9]. VSX1 is essential for craniofacial and ocular development, and it has been reported that VSX1 mutations play a pathogenic role in posterior polymorphous corneal dystrophy [10–12]. However, few studies have focused on the oncogenic role of VSX1. A study involving quantitative genome-wide methylation analyses in bladder cancer reported that higher methylation frequencies in VSX1 were positively associated with the risk of high-grade non-muscle invasive bladder cancer [13]. Therefore, there is an urgent need to investigate the oncogenic role of VSX1 in the tumorigenesis, progression, and metastasis of ccRCC.

FK506-binding protein 10 (*FKBP10*), a member of the FKBP subfamily immunophilins, is a chaperone that directly interacts with collagen I [14]. In recent studies, an oncogenic role of *FKBP10* has emerged. For example, overexpression of *FKBP10* promoted lung cancer growth in vitro or in vivo, indicating that this gene might be a putative therapeutic target for lung cancer [15, 16]. Additionally, *FKBP10*, which drove colorectal cancer progression and was associated with a poor prognosis, might be an essential prognostic risk factor [17, 18]. Furthermore, *FKBP10* was screened and identified as a novel potential biomarker for breast cancer brain metastasis [19]. However, the upstream regulatory mechanisms of *FKBP10* have yet to be investigated.

In this study, VSX1 was significantly upregulated in ccRCC tissues and was associated with a poor prognosis of the disease. Overexpression of VSX1 induced proliferation, invasion, and migration of ccRCC cells. Regarding the mechanism underlying these activities, VSX1 could bind to the promoter of *FKBP10* to upregulate its transcription and promote the tumorigenesis and progression of ccRCC. Findings from the study suggested that targeting VSX1, an important prognostic biomarker, might prevent ccRCC progression.

## Methods

### Data collection and bioinformatics analysis

RNA-sequence data were downloaded from TCGA and one Gene Expression Omnibus (GEO) dataset. Clinical data were downloaded from the TCGA database (https://portal.gdc.cancer.gov/). The survival prognosis of related genes was investigated via the Kaplan–Meier method using 539 samples of ccRCC in TCGA, with analsyses including overall survival (OS), disease-specific survival (DSS), and progression-free interval (PFI). Kaplan–Meier curves of survival analyses with log-rank *P* values < 0.05 were drawn. The group cutoff was set as 50% of VSX1 expression. Using a hypothetical two-tailed test, differentially expressed genes (DEGs) between the high- and low-expressed VSX1 groups were identified using the *R* package, “DESeq2” [20]. Thresholds were set as a log-fold change > 1.5 and an adjusted *P*-value < 0.05. Based on Spearman’s test, a preranked list of DEGs was ordered by the relationship with VSX1 using the *R* package “ggplot2” [21]. In addition, an enrichment analysis was conducted using the *R* package, “ClusterProfiler” to identify VSX1-related molecular mechanisms in DEGs [22]. Gene Ontology (GO) functions and the Kyoto Encyclopedia of Genes and Genomes (KEGG) pathways were analyzed to determine the top 100 up- and down-regulated DEGs. The *R* package, “pheatmap”, was applied to present gene expression (*BEST4*, *LMO1*, *ARID3C*, *TEME44*, *FKBP10*, *TRIB3*) in all ccRCC samples as heatmaps. Pearson’s test was used to verify the relationship of these six DEGs with VSX1, and the results were visualized as scatter plots. To investigate the prognostic benefits of six co-expressed DEGs (*BEST4*, *LMO1*, *ARID3C*, *TEME44*, *FKBP10*, *TRIB3*) in OS, DSS, and PFI, univariate Cox-regression analyses were employed based on the “survival” and “survminer” *R* packages. A forest plot was used to examine the correlation between the six co-expressed DEGs and prognosis in T stage subgroups and pathological stage subgroups of ccRCC using the *R* package “forestplot”. To validate the diagnostic efficiency of these six DEGs, receiver operating characteristic (ROC) curves were built and the area-under-the-curve (AUC) value of each gene was calculated by using the *R* package “pROC” [23]. The prognostic significance of VSX1 in ccRCC was revealed, and the expression level of VSX1 was positively associated with the T stage, lymph node stage, and pathological stage of the disease. Similarly, the expression level of VSX1 was also investigated in the pan-cancer dataset from TCGA using an online tool, Xiantao Academy (https://www.xiantao.love), which consists of multiple kinds of tumor and adjacent tissue samples. The mRNA relative expression level of VSX1 was comparatively analyzed between the adjacent and tumoral tissues based on GSE40435 (http://ncbi.nlm.nih.gov/geo/).

### Clinical samples collection

Frozen ccRCC tissues and matched normal adjacent tissues for quantitative real-time PCR (n = 20), and paraffin-embedded cancerous tissues (n = 40) and peritumoral kidney tissue microarrays (n = 19) for immunohistochemistry (IHC), were collected from patients undergoing surgery in the Affiliated Drum Tower Hospital, Medical School of Nanjing University. All patients enrolled in the study were diagnosed with ccRCC according to the World Health Organisation (WHO) classification system, and pathological diagnoses were reviewed retrospectively. Clinical and pathological data were collected from patients and their informed consent to participate in the study was obtained.

### Cell lines and cell culture

Human RCC cell lines in the study included the renal adenocarcinoma cell line 786-O (ATCC, CRL-1932), renal adenocarcinoma cell line 769-P (ATCC, CRL-1933), renal adenocarcinoma cell line ACHN (ATCC, CRL-1611), renal carcinoma cell line A498, clear cell renal carcinoma cell line Caki-1, kidney cortex/proximal tubule cell line HK-2 (ATCC, CRL-2190) and embryonic kidney cell line HEK-293T (ATCC, CRL-3216). The human renal carcinoma cell line A498 was purchased from the National Infrastructure of Cell Line Resource (Beijing, China). The ccRCC cell line Caki-1 was obtained from the Cell Bank of the Chinese Academy of Science (Shanghai, China). These cells, except for Caki-1, were cultured in DMEM (Wisent, 31900608) supplemented with 10% fetal bovine serum (FBS) (Gibco, 2232510). Caki-1 cells were cultured in McCoy’s 5A medium (Gibco, 16600082), containing 10% FBS (Gibco, 2232510). All media contained 1% penicillin and streptomycin (Gibco, 15140122). All cell lines were incubated at 37 °C with 5% CO_2_.

### Western blotting

Total protein was extracted from cells for western blotting. Cells were lysed with moderate RIPA lysis buffer (Beyotime, P0013C) after rinsing three times in PBS at 4 °C and were centrifuged. The resulting supernatant was mixed with loading buffer and heated at 100 °C for 10 min. Proteins were separated by sodium dodecyl sulfate–polyacrylamide gel electrophoresis (SDS-PAGE) and transferred to a polyvinylidene fluoride (PVDF) membrane (Roche, Basel, Switzerland) and blocked in 5% bovine serum albumin (BSA) (SigmaAldrich) for 1 h at room temperature. Blots were then incubated with primary antibodies at 4 °C overnight, and secondary antibodies conjugated with horseradish peroxidase were incubated for 1 h at room temperature. Protein signals were detected with ECL solution (Millipore) and quantified with Image J software (National Institutes of Health). β-actin (ACTB) was used as an internal control.

### RNA isolation and quantitative real-time PCR (qRT-PCR) assay

Total RNA was isolated using the RNA-easy isolation reagent (Vazyme, R701-01) according to the manufacturer’s instructions and then reverse-transcribed into cDNA with HiScript Q RT SuperMix for qPCR (Vazyme, R122-01). Subsequently, quantitative real-time PCR was performed to quantify cDNA using Taq Pro Universal SYBR qPCR Master Mix (Vazyme, Q712-02). For normalization, 18S rRNA was used as an internal reference, and 2^−ΔΔCt^ was used to analyze the relative expression levels of target genes. Primer sequences for target genes were listed in Additional file 1: Table S1.

### Immunohistochemical (IHC) staining and scoring

ParafFin-embedded tissue microarrays were dewaxed using dimethyl benzene and rehydrated with gradient ethanol solutions. Then, 3% H_2_O_2_ was used to inhibit the endogenous peroxidase for 15 min, and the antigens were retrieved by microwave heating. The antigens were blocked with 10% BSA for 1 h. The tissue microarrays were incubated with the primary antibody against VSX1 (1:300; LS-C829216, Life science) at 4 °C overnight and then incubated with the secondary antibody in the dark for 2 h. Diaminobenzidine was used to visualize immunostaining. The staining intensity of VSX1 was quantified with Image J software.

### CCK-8 cell proliferation assay and clone formation assay

The indicated cells were seeded in the 96-well plates (3 × 10^3^ cells/well) and cultured overnight. The next day, cell proliferation was evaluated using the Cell Counting Kit-8 reagent (CCK-8, MCE), and the absorbance values were measured at 450 nm. Thereafter, 1 × 10^3^ RCC cells were seeded into 6-well plates and cultured for 2 weeks. The number of cell clones formed was assessed after crystal violet (Beyotime, C0121) staining, which was imaged and counted under a microscope.

### Transwell assay

Cell migration and invasion experiments were performed using a Transwell chamber (Corning, USA). After serum-starving the cells for 24 h, 1 × 10^5^ cells with 200 μL serum-free medium were seeded into the upper chamber, and the lower chamber was filled with 500 μL DMEM (Wisent, 31900608) supplemented with 10% FBS (Gibco, 2232510). The migrated cells were imaged and counted under a microscope after crystal violet (Beyotime, C0121).

### Flow cytometry

Flow cytometry was conducted based on the manufacturer’s instructions. Cells were measured with a BD Beckman cytometer (BD Biosciences) and analyzed using FlowJo software after incubating with the Annexin V-PE/7-ADD Apoptosis Detection Kit (Vazyme, A213-01). Similarly, cells were incubated with the PI/RNase Staining Kit (BD Biosciences) to analyze the cell cycle and were quantified with a BD Beckman cytometer.

### Dual‑luciferase reporter assay

The pGL3-Basic plasmids-containing promoter region of the target gene and VSX1 overexpression plasmid was constructed and transfected into HEK-293T cells. The pRL-TK-Renilla luciferase plasmid was transfected into the same cells and used as an internal reference. The dual-Luciferase Reporter Assay Kit (Vazyme, DL101-01) was used to evaluate the activities of firefly luciferase and Renilla luciferase. The pGL3-Basic vector plasmid was used as an internal control for reporter genes.

### Tumor sphere formation

For the tumor sphere formation assay, 1 × 10^3^ Caki-1 or 786-O cells were seeded into U-bottom ultra-low attachment 96-well plates (Corning, cat. no. 174925) and cultured in DMEM supplemented with 10% FBS for 2 weeks. Tumor sphere images were captured under a microscope and the area was measured with Image J software (National Institutes of Health).

### Plasmid construction, lentivirus, and cell transfection

The VSX1 overexpression plasmid ligated with the linearized TK-PCDH-copGFP-T2A-Puro vector plasmid was constructed by Tsingke Technology (Beijing, China). Promoter sequences for the target genes—*TMEM44*, *FKBP10*, and *TRIB3*—were acquired from the National Center for Biotechnology Information (NCBI; https://www.ncbi.nlm.nih.gov/), and the purified promoter fragments were ligated with the linearized pGL3Basic vector plasmid. The sequences of VSX1-shRNA and *FKBP10*-shRNA were obtained from Sigma (https://www.sigmaaldrich.cn/CN/zh) and ligated with pLVshRNA-EGFP-Puro vector plasmid. All targeted sequences were synthesized by Tsingke Technology (Beijing, China). Cells were transfected with shRNA or plasmids as per the manufacturer’s protocols of jetPRIME (Polyplus-transfection, Shanghai, Illkirch, France). Cells were harvested 48 h after transfection. The shRNA sequences are listed in Additional file 1: Table S1.

### Statistical analysis

Data were expressed as the means ± standard deviation. SPSS 23.0 software (SPSS Inc., Chicago, IL) was used for statistical analyses of the data. GraphPad Prism 8.0 (GraphPad Software, San Diego, CA, USA) was used to depict Kaplan–Meier survival curves and compare between subgroups using the log-rank test. Spearman’s correlation coefficient was calculated to analyze the connection between VSX1 expression and that of other target genes. Student’s *t*-test and one-way analysis of variance (ANOVA) were used to assess the significance of differences; *P* < 0.05 was considered statistically significant (*P < 0.05, ** *P* < 0.01, and *** *P* < 0.001).

## Results

### VSX1 was identified as a novel prognostic biomarker of ccRCC

**Fig. 1:**
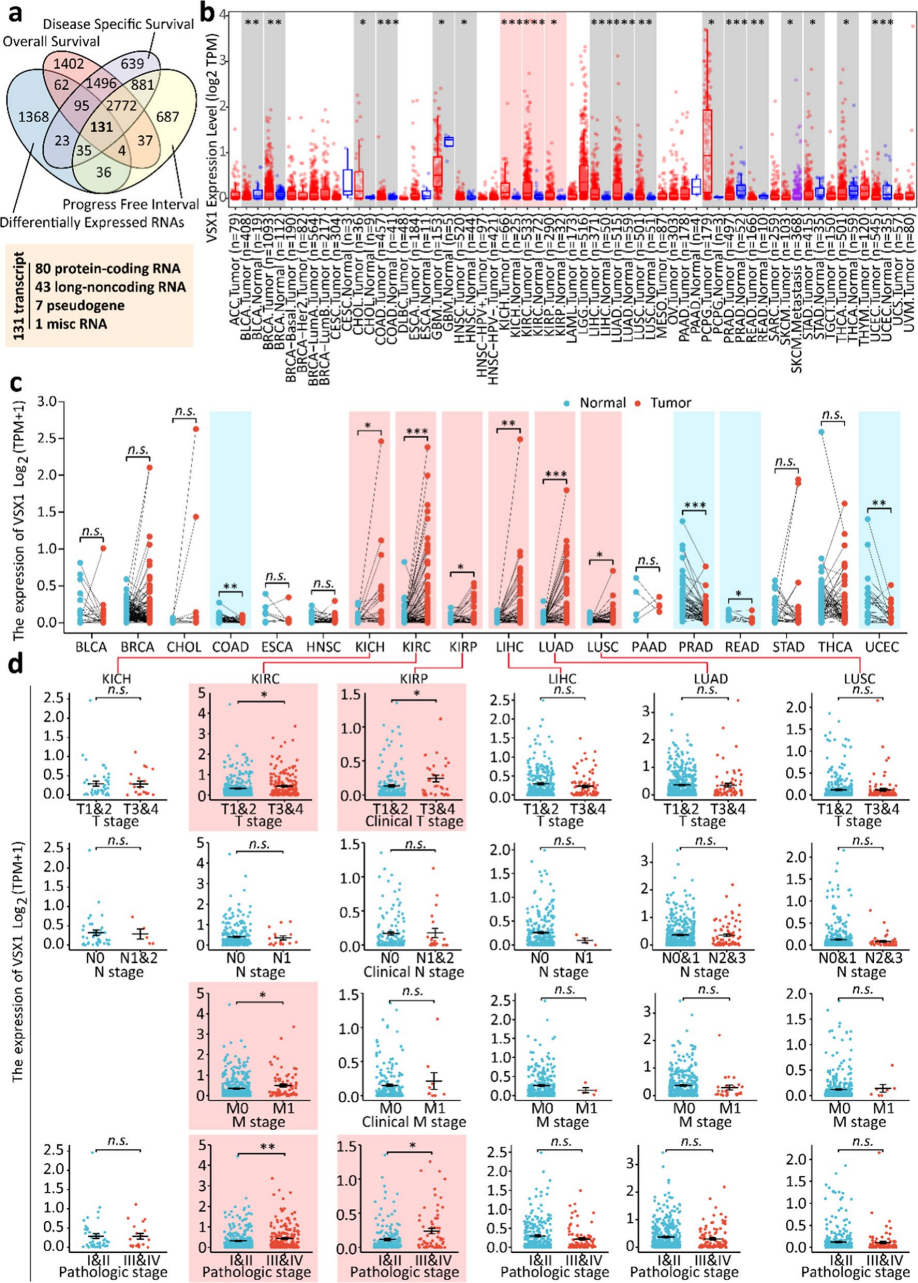
Identification of VSX1 as a prognostic biomarker in ccRCC. **a** Venn-diagram analyses of candidate genes from the TCGA database. **b** VSX1 mRNA expression in diverse human malignancies. Statistical significance was calculated using the Wilcoxon test. **c** VSX1 mRNA expression was higher in ccRCC tissue than in the adjacent normal tissue in the TCGA database. The significance of the difference was evaluated using the paired Student’s *t*-test. **d** VSX1 mRNA expression was analyzed in different pathological stages for ccRCC in the TCGA database. The significance of the difference was evaluated using unpaired Student’s *t*-test. *P < 0.05, ** *P* < 0.01, and *** *P* < 0.001

**Fig. 2:**
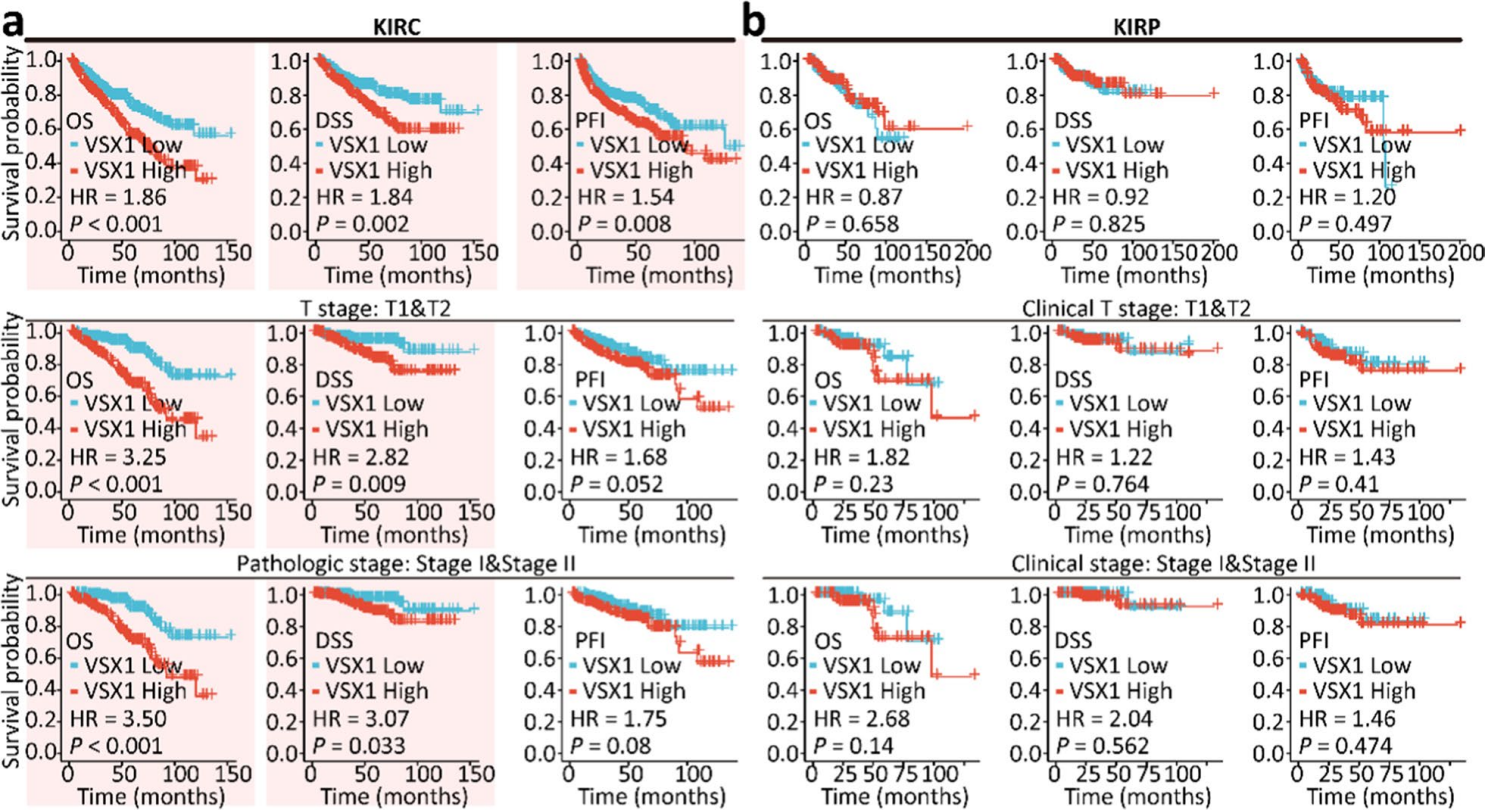
Survival analyses for ccRCC and pRCC patients using the Kaplan–Meier method. **a** High VSX1 expression was correlated with poor overall survival (OS), disease-specific survival (DSS), and progression-free interval (PFI) in ccRCC. **b** OS, DSS, and PFI for pRCC patients with low or high levels of VSX1 in the TCGA database. Kaplan–Meier methods of survival curves with log-rank *P*-value < 0.05 were drawn. The group cutoff was set as 50% of the VSX1 expression

To search for novel prognostic biomarkers of ccRCC, the survival prognosis, including OS, DSS, and PFI, of related genes was investigated for patients with ccRCC. Differentially expressed target genes were identified between ccRCC and peritumoral tissues. A four-way Venn diagram showed there were 131 DEGs in ccRCC tissues compared with peritumoral tissues (Fig. 1a and Additional file 2: Table S2). Among these genes, 80 were potential protein-coding genes. A total of 56 candidate upregulated genes were identified and were listed in Additional file 2: Table S2. However, some of these genes, such as *RNASET2*, *TF*, and *ZIC2*, were already known to have an oncogenic role in the tumorigenesis, progression, and metastasis of renal cancer [24–26]. Among these candidate upregulated genes, VSX1 was selected and further analysis focused on the oncogenic role of VSX1 in ccRCC. Figure 1b showed that VSX1 mRNA was not only upregulated in cancerous tissues of ccRCC, but was also upregulated in pan-cancer tissues. In addition, the expression level of VSX1 in ccRCC tissues was higher than that in the matched normal adjacent tissues (Fig. 1c). Clinical correlation studies of data revealed that upregulated VSX1 mRNA was strongly associated with advanced T stages, distant metastasis, and high pathological stages. However, no such correlation was found with lymphatic metastasis (Fig. 1d). Similarly, the VSX1 mRNA level and an advanced T stage or a high pathological stage were associated with papillary RCC (pRCC) (Fig. 1d). Additionally, the survival analyses revealed that OS, DSS, and PFS were shortened for ccRCC patients with a high expression of VSX1 (Fig. 2a). However, no prognostic difference was observed in pRCC patients (Fig. 2b). These data implied that the upregulation of VSX1 might play an essential role in the progression of ccRCC.

### The upregulation of VSX1 was validated in ccRCC clinical samples and cell lines

**Fig. 3:**
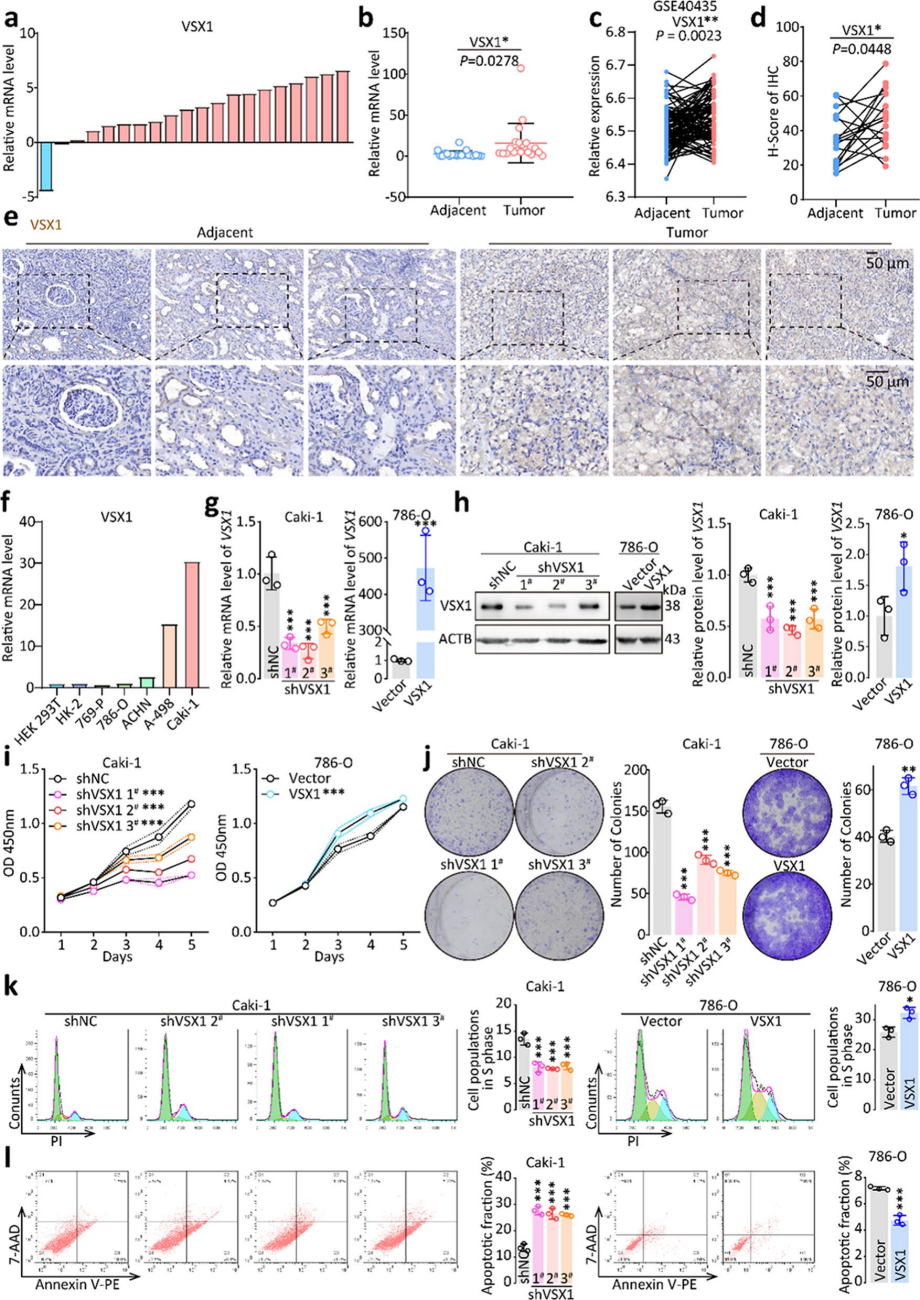
High VSX1 expression promoted cancer cell proliferation, invasion, and migration in ccRCC. **a** − **b** qRT-PCR analyses of relative VSX1 mRNA expression in 20 pairs of human clinical ccRCC tissues. **c** VSX1 mRNA expression was validated based on the GSE40435 dataset. **d** − **e** Representative IHC staining of VSX1 in ccRCC tissues compared with paired adjacent noncancerous tissues and the staining scoring analysis. **f** VSX1 mRNA expression levels in a panel of human RCC cell lines were detected by qRT-PCR. **g** − **h** qRT-PCR and western blot verified the overexpression of VSX1 in 786-O cells and the knockdown of VSX1 in Caki-1 cells. **i** CCK-8 evaluated cell proliferation following VSX1 overexpression or knockdown. **j** VSX1 knockdown in Caki-1 cells inhibited colony formation, whereas the opposite results were reported in 786-O cells overexpressing VSX1. **k** − **l** Flow cytometry analyses showed that the knockdown of VSX1 expression in Caki-1 cells significantly increased the apoptosis rate and reduced cell populations in the S phase. Paired or unpaired Student’s *t*-tests and the one-way analysis of variance were used to assess the significance of differences. Data were presented as the mean ± standard deviation.*P < 0.05, ** *P* < 0.01, and *** *P* < 0.001

After screening VSX1 as a potential prognostic biomarker by using bioinformatics methods, VSX1 mRNA levels were detected in 20 pairs of ccRCC tissues and adjacent nontumoral tissues by qRT-PCR. Of the 20 cancerous samples, 19 (95%) had upregulated VSX1 mRNA levels compared with adjacent noncancerous tissues (*P* = 0.0278, Fig. 3a and b). The upregulation of VSX1 was consistently confirmed between the adjacent and tumoral tissues based on dataset GSE40435 from the GEO database (*P* = 0.0023, Fig. 3c). Furthermore, the upregulation of VSX1 was also confirmed in cancerous tissues by the IHC assay. IHC scoring showed that the expression of VSX1 increased in cancerous tissues compared with normal kidney tissues (Fig. 3d and e).

The expression of VSX1 was further detected in HEK-293 T cells, the immortal kidney cortex/proximal tubule cell line HK-2, and a panel of human RCC cell lines by qRT-PCR. Human RCC cells (786-O, 769-P, ACHN, A498, and Caki-1) exhibited higher expression of VSX1 compared with HEK-293 T and HK-2 cells, with A498 and Caki-1 cells showing the highest expression (Fig. 3f). The Caki-1 cell line was acquired from metastatic sites of the skin, while the A498 cell line was initially obtained from the primary tumor of a female patient.

### Knockdown or overexpression of VSX1 affected ccRCC aggressiveness in vitro

To reveal the oncogenic role of VSX1 upregulation in ccRCC progression, VSX1 was overexpressed in the ccRCC cell line 786-O and knocked down in the Caki-1 cell line. Stable VSX1 overexpression or knockdown cell lines were constructed using a lentivirus system and confirmed by qRT-PCR and western blotting analyses (Fig. 3g and h).

In the CCK-8 assay, 786-O cells with stable VSX1 overexpression displayed significantly enhanced growth compared with that in the empty vector control group (Fig. 3i). In contrast, VSX1 knockdown in Caki-1 cells markedly suppressed cell proliferation compared with the shRNA negative control (shNC) group. Moreover, colony formation assays indicated that VSX1 knockdown in Caki-1 cells inhibited colony formation; the opposite results were revealed in 786-O cells overexpressing VSX1 compared with the empty vector control group (Fig. 3j). Furthermore, a cell cycle assay showed a marked reduction of cell populations in the S phase induced by the knockdown of VSX1 in Caki-1 cells, and the opposite results were obtained in 786-O cells with overexpression of VSX1 in comparison with the shNC group (Fig. 3k). This result was also confirmed by the CCK-8 assay.

Flow cytometry analyses implied that the knockdown of VSX1 expression significantly increased the apoptosis rate in ccRCC cells (Fig. 3l), and opposing results were obtained in 786-O cells overexpressing VSX1 compared with the empty vector control group. Moreover, the tumor sphere formation assay revealed that knockdown of VSX1 expression in Caki-1 cells significantly inhibited the tumor sphere formation capacity of the cells compared with that of the shNC group (Additional file 3: Fig. S1a). Conversely, compared with the empty vector control group, the overexpression of VSX1 in 786-O cells markedly enhanced tumor sphere formation ability of the cells (Additional file 3: Fig. S1b). The Transwell assay, performed to evaluate the migration and invasion abilities of ccRCC cells, revealed that upregulation of VSX1 promoted the migration and invasion abilities of Caki-1 cells and 786-O cells, and vice versa (Additional file 3: Fig. S1c and d). Collectively, these findings suggested that VSX1 acted as a tumor activator gene in ccRCC.

### Gene correlation analyses revealed VSX1 was an essential upstream transcriptional regulator

**Fig. 4:**
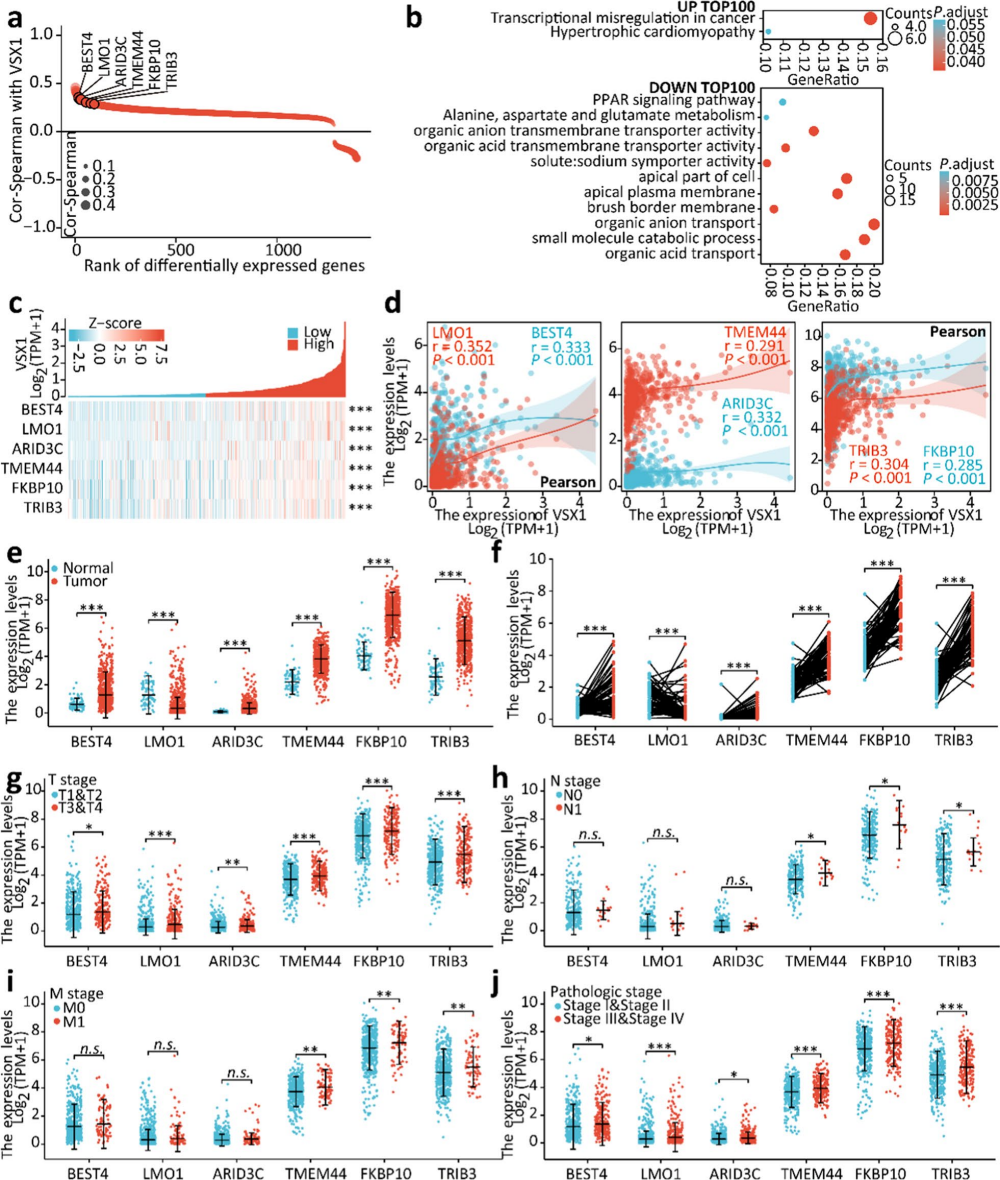
Gene correlation analyses suggested that VSX1 might be an essential upstream transcriptional regulator. **a** Gene correlation analyses between VSX1 and other differentially expressed genes (DEGs) in ccRCC from TCGA via Spearman’s correlation coefficient. **b** GO and KEGG pathway analyses were conducted using VSX1-correlated genes. **c** − **d** The expression levels of VSX1-correlated genes positively correlated with upregulated VSX1 by heatmap and Pearson’s correlation analyses. **e** − **f** The expression level of VSX1-correlated genes was higher in ccRCC tissue in the TCGA database. **g** − **j** mRNA expression of VSX1-correlated genes was analyzed in different pathological stages for ccRCC in the TCGA database. Paired or unpaired Student’s *t*-tests were used to assess the significance of differences. Spearman’s test and Pearson’s test were used to verify the relationship between DEGs and VSX1. Data were presented as the mean ± standard deviation.*P < 0.05, ** *P* < 0.01, and *** *P* < 0.001

Gene correlation analyses were performed between VSX1 and other DEGs in ccRCC from TCGA using the Spearman’s correlation coefficient. As shown in Fig. 4a, many upregulated or downregulated genes in ccRCC were correlated with the expression of VSX1. KEGG analysis via a bubble chart of the top 100 up- or down-regulated genes indicated that these genes showed good correlation with VSX1 and might play a leading role in the transcriptional misregulation of cancer and organic acid transmembrane transporter activity (Fig. 4b).

Since VSX1 is essentially a transcription factor [27], we focused on the top upregulated genes relevant to transcriptional dysregulation, such as *BEST4*, *LMO1*, *ARID3C*, *TMEM44*, *FKBP10*, and *TRIB3*. Pearson’s correlation analyses suggested that the expression levels of *BEST4*, *LMO1*, *ARID3C*, *TMEM44*, *FKBP10*, and *TRIB3* were positively correlated with upregulated VSX1, which was also observed in the heatmap analysis (Fig. 4c and d). Examination of the expression of these genes in ccRCC samples revealed that mRNA expression of these genes was higher in cancerous tissues than in the normal adjacent tissues, and this finding was confirmed in the matched normal and cancerous tissues (Fig. 4e and f).

**Fig. 5:**
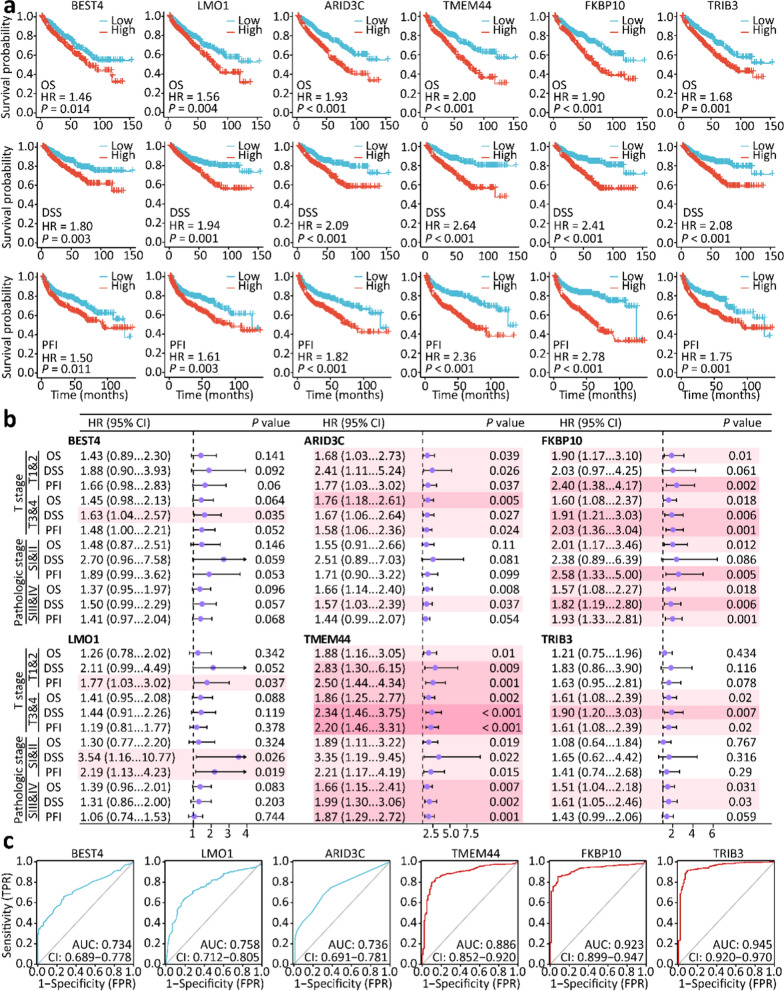
The identification of VSX1-correlated genes as prognostic biomarkers of ccRCC. **a** Kaplan − Meier analysis of overall survival (OS), disease-specific survival (DSS), and progression-free interval (PFI) for ccRCC patients with VSX1-correlated genes in the TCGA database. **b** Forest plot analyses of VSX1-correlated genes as prognostic risk factors for patients with different pathological stages of ccRCC. **c** VSX1-correlated genes showed a high accuracy in predicting normal and cancer outcomes via ROC curves

The expression of these genes in ccRCC samples was further investigated in relation to the T stage, N stage, M stage, and pathological stage. As shown in Fig. 4g–j, upregulated mRNA levels of *BEST4*, *LMO1*, and *ARID3C* were associated with an advanced T stage and a high pathological stage; however, the association was not found in the N stage and M stage. In addition, upregulated mRNA levels of *TMEM44*, *FKBP10*, and *TRIB3* were strongly associated with advanced T stage, lymphatic metastasis, distant metastasis, and a high pathological stage. Survival analyses implied that OS, DSS, and PFI were shortened for ccRCC patients with high expression of these genes (Fig. 5a). Moreover, high expression of *TMEM44*, *FKBP10*, and *TRIB3* was an unfavorable prognostic risk factor for ccRCC patients in advanced T and high pathological stages (Fig. 5b). These genes might therefore have potential as diagnostic biological markers for ccRCC (Fig. 5c). These data led to the reasonable assumption that VSX1 might transcriptionally regulate these tumor-related genes either directly or indirectly, and this hypothesis warranted further experimental verification.

### VSX1 affected ccRCC invasiveness through transcriptionally regulating FKBP10

**Fig. 6:**
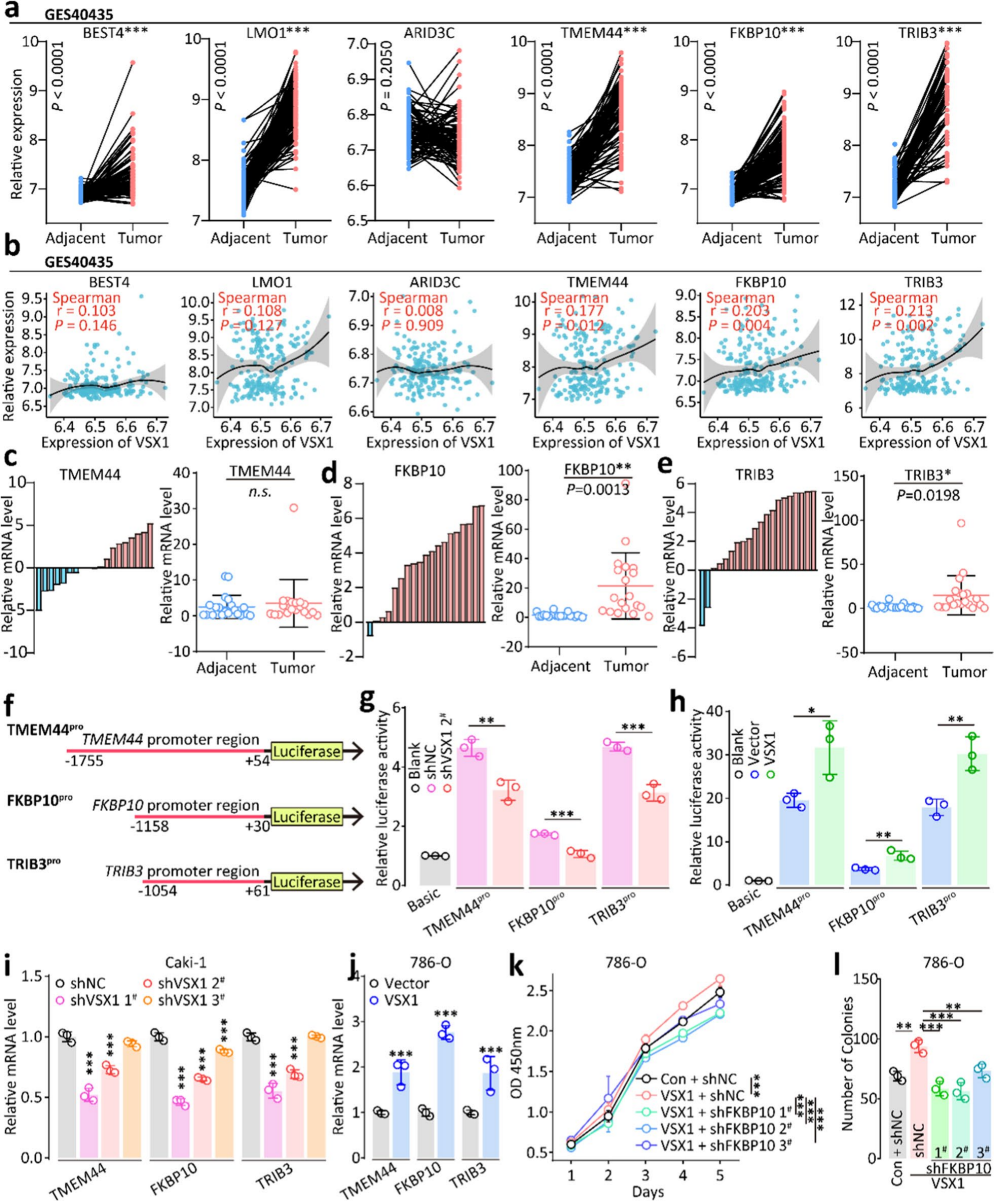
High VSX1 expression promoted tumor invasion in ccRCC by transcriptional activation. **a** The mRNA expression of VSX1-correlated genes was validated in the GSE40435 dataset. **b** The gene-correlation analysis was validated on GSE40435 by Spearman’s correlation coefficient. **c** − **e** qRT-PCR assay of relative mRNA expression of *TMEM44*, *FKBP10*, and *TRIB3* in 20 pairs of clinical ccRCC tissues. **f** − **h** Dual-luciferase reporter gene analysis of transcriptional activation for VSX1. **i** − **j** qRT-PCR analysis of relative mRNA expression of *TMEM44*, *FKBP10*, and *TRIB3* in 786-O and Caki-1 cells. **k** − **l** CCK-8 and colony formation evaluated the proliferation capacity for *FKBP10* knockdown after VSX1 overexpression in 786-O cells. Paired or unpaired Student’s *t*-tests and one-way analysis of variance were used to assess the significance of differences. Data were presented as the mean ± standard deviation. *P < 0.05, ** *P* < 0.01, and *** *P* < 0.001

The upregulation of *TMEM44*, *FKBP10*, and *TRIB3* was confirmed between the adjacent and tumoral tissues based on dataset GSE40435 from the GEO database (Fig. 6a). Gene correlation analyses between VSX1 and *TMEM44*, *FKBP10*, or *TRIB3* were also performed based on dataset GSE40435. *TMEM44*, *FKBP10*, and *TRIB3* were positively correlated with VSX1 (Fig. 6b). Next, the expression of these genes was detected by qRT-PCR in 20 pairs of ccRCC tissues and adjacent nontumoral tissues. There was no significant difference in the expression of *TMEM44* between cancerous samples and adjacent noncancerous tissues (Fig. 6c), but both *FKBP10* and *TRIB3* mRNA were upreglated in most cancerous samples (Fig. 6d and e).

The dual-luciferase reporter gene assay revealed that the knockdown of VSX1 expression significantly decreased the relative luciferase activities of *TMEM44*, *FKBP10*, and *TRIB3* (Fig. 6g), while the overexpression of VSX1 increased the relative luciferase activities of these genes (Fig. 6h). Next, VSX1 protein levels were changed by either knockdown or overexpression in vitro, and the expression levels of *TMEM44*, *FKBP10*, and *TRIB3* in Caki-1 and 786-O cells were analyzed via qRT-PCR, respectively. The expression of *TMEM44*, *FKBP10*, and *TRIB3* was downregulated by the knockdown of VSX1 compared with the negative control in Caki-1 cells (Fig. 6i), and similar results were confirmed in 786-O cells (Fig. 6j). The CCK-8 assay showed that significantly enhanced growth in 786-O cells with stable VSX1 overexpression was almost restored to normal by the knockdown of *FKBP10* (Fig. 6k).

The CCK-8 assay showed significantly inhibited growth in Caki-1 cells with stable *FKBP10* knockdown (Additional file 4: Fig. S2a), while the Transwell assay demonstrated that the knockdown of *FKBP10* inhibited the migration and invasion abilities of Caki-1 cells (Additional file 4: Fig. S2b and c). Colony formation assays revealed that *FKBP10* knockdown in 786-O cells inhibited colony formation (Fig. 6l and Additional file 4: Fig. S2d). In addition, the tumor sphere formation assay indicated that the ability to inhibit the tumor sphere formation in 786-O cells with the knockdown of *FKBP10* after stable VSX1 overexpression was stronger than that in the shNC group (Additional file 4: Fig. S2e and f). A Transwell assay was performed to illuminate the impact of *FKBP10* on the behavior of 786-O cells and showed that downregulation of *FKBP10* inhibited the migration and invasion ability of the cells compared with the shNC group (Additional file 4: Fig. S2g and h). These findings suggested that VSX1 affected tumor invasiveness via transcriptionally regulating *FKBP10*.

## Discussion

CCRCC, the most common malignant kidney neoplasm, accounts for 60 − 70% of all new RCC cases and deaths worldwide [28]. Despite considerable development in surgical plans and systemic therapies for ccRCC, the clinical efficacy of these therapeutic options for locally advanced and metastatic ccRCC is still substandard [29–31]. Thus, there is an urgent need to explore effective biomarkers and novel molecular mechanisms underlying ccRCC and develop more effective anti-tumor therapies. In the present study, we screened the TCGA database to identify VSX1 as a novel oncogenic activator in ccRCC and demonstrated that VSX1 affected ccRCC aggressiveness in vitro. VSX1 was upregulated in ccRCC tissues from the GEO and TCGA databases as well as in clinical cancerous specimens from our department compared with adjacent noncancerous tissues. Additionally, upregulated VSX1 mRNA was strongly associated with an advanced T stage, distant metastasis, and a high pathological stage, proving to be a significantly effective prognostic biomarker in patients with ccRCC.

Recent studies reported that VSX1 mutations played a pathogenic role in posterior polymorphous corneal dystrophy [32, 33], and that the variant p.(His244Arg) in the VSX1 gene was observed in a sporadic female patient with bilateral keratoconus [34]. However, there are limited investigations focusing on the relationship between VSX1 upregulation and tumor aggressiveness. A recent study indicated that higher methylation frequencies in VSX1 had a significantly positive association with the risk of the high-grade non-muscle invasive bladder cancer [13], but the role of the VSX1 gene in other human cancers has not been confirmed. Thus, we demonstrated for the first time that the upregulation of VSX1 affected ccRCC aggressiveness.

A previous study reported that VSX1 protein expression was retina-specific in adult mouse tissues, and mouse VSX1 and human VSX1 were divergent, sharing a 71% overall amino acid identity [35]. Many studies have confirmed that the VSX1 protein is expressed in the human retina and mutated in various eye diseases [9, 12, 36]. In this study, we confirmed detectable VSX1 protein expression in ccRCC tissues, but it was hardly expressed in normal kidney tissues. Therefore, the mechanism by which VSX1 protein is expressed in ccRCC tissues needs further exploration.

As a transcriptional factor, VSX1 can be either a transcriptional repressor or a transcriptional activator. For example, VSX1 can function as a transcriptional repressor in the terminal differentiation of a subset of bipolar cells, and a VSX1 mutation that impairs DNA binding and causes keratoconus in humans hindered repressor function [9, 37]. In this study, VSX1 was confirmed to function in an oncogenic role as a transcriptional activator in ccRCC. However, the mechanism by which VSX1 mediates the expression of downstream target genes and regulates the invasiveness of ccRCC requires further investigation.

To determine the downstream target genes of VSX1, we performed gene correlation analyses between VSX1 and other differentially expressed genes in ccRCC from the TCGA database and a GEO dataset. The genes—including *BEST4*, *LMO1*, *ARID3C*, *TMEM44*, *FKBP10*, and *TRIB3—*were correlated with VSX1 and might play a primary role in the transcriptional misregulation of cancer from the TCGA database. *BEST4* belongs to the bestrophin protein family that is widely expressed in human intestinal epithelial cells. Recent studies reported the oncogenic potential of *BEST4* in colorectal carcinogenesis and metastasis by modulating PI3K/Akt signaling [38, 39]. Although *BEST4* was upregulated in ccRCC tissues compared with normal kidney tissues, based on the TCGA database, a good correlation with VSX1 was not observed in the GEO dataset. Similarly, *LMO1* and *ARID3C* also displayed a poor correlation with VSX1 in the dataset from the GEO database. Moreover, upregulated mRNA levels of *BEST4*, *LMO1*, and *ARID3C* were associated with the advanced T stage and a high pathological stage; however, these associations were not found in the N stage and M stage. Therefore, we focused on *TMEM44*, *FKBP10*, and *TRIB3*, which were strongly associated with an advanced T stage, lymphatic metastasis, distant metastasis, and high pathological stage. *TMEM44* is an integral component of membrane proteins, and previous studies demonstrated that upregulated lncRNA TMEM44-AS1 might promote glioma progression and was correlated with 5-FU resistance in gastric cancer [40, 41]. Although no difference in *TMEM44* expression was detected between ccRCC tissues and the adjacent normal kidney tissues, *TMEM44* was still likely one of the downstream target genes of VSX1. However, *FKBP10* was upregulated in ccRCC tissues compared with the normal kidney tissues from our department, and this was also likely owing to transcriptional regulation by VSX1. Consistently, previous studies have demonstrated that *FKBP10* plays an oncogenic role in many human malignancies. For example, circREEP3 upregulation recruited the chromatin remodeling protein CHD7 to activate *FKBP10* transcription and drive colorectal cancer progression [17], and the *FKBP10* protein was recognized as a glioma antigen for anti-glioma mRNA vaccine production [42]. In addition, *FKBP10* promoted tumor cell proliferation, invasion, and migration in stomach adenocarcinoma [43], and similar findings were observed in ccRCC in this study.

*TRIB3* is a protein kinase that controls cell proliferation and differentiation and has been demonstrated to promote cancer development [44]. *TRIB3* is positively associated with breast cancer stemness and progression by inhibiting FOXO1 degradation and increasing SOX2 transcription [45] and the cellular stress-activated TRB3/USP9x-dependent Notch pathway in breast cancer [46]. Our study demonstrated that *TRIB3* upregulation occurred in ccRCC tissues and was regulated transcriptionally by VSX1; the detailed mechanism of this process merits a more profound investigation.

## Conclusion

VSX1 mRNA upregulation was generally observed in multiple human malignancies from the TCGA database and was confirmed in both a GEO dataset and ccRCC clinical specimens from our department. Survival analyses identified VSX1 as a novel prognostic biomarker in ccRCC. The in vitro experiments confirmed that the overexpression of VSX1 promoted ccRCC aggressiveness. Mechanistically, high expression of VSX1 promoted cancer cell proliferation, invasion, and migration in ccRCC via transcriptional activation of downstream target genes.

## Supplementary Information

**Additional file 1: Table S1.** Primers sequence used for quantitative real-time PCR and shRNA sequence.

**Additional file 2: Table S2.** Differentially expressed genes were listed.

**Additional file 3: Fig. S1.** Verification of the impact of VSX1 on *tumor* cell invasiveness. (a−b) Tumor sphere formation assays evaluated the sphere-forming capacity for VSX1 overexpression or knockdown in 786-O and Caki-1 cells. (c−d) The Transwell assay evaluated cell migration and invasion of 786-O and Caki-1 cells with VSX1 overexpression or knockdown. Unpaired Student’s *t*-tests were used to assess the significance of differences. Data were presented as the mean ± standard deviation.*P < 0.05, ** *P* < 0.01, and *** *P* < 0.001.

**Additional file 4: Fig. S2.** Verification of the impact of VSX1 transcriptional activation on *tumor *cell invasiveness. (a) CCK-8 evaluated the proliferation of Caki-1 cells following *FKBP10* knockdown. (b−c) The Transwell assay evaluated cell migration and invasion for *FKBP10* knockdown in Caki-1 cells. (d) Representative colony formation of 786-O cells for *FKBP10* knockdown after VSX1 overexpression. (e−f) The tumor sphere formation assay evaluated sphere-forming capacity of *FKBP10* knockdown after VSX1 overexpression in 786-O cells. (g−h) The Transwell assay evaluated cell migration and invasion for *FKBP10* knockdown after VSX1 overexpression in 786-O cells. Unpaired Student’s *t*-tests were used to assess the significance of differences. Data were presented as the mean ± standard deviation. *P < 0.05, ** *P* < 0.01, and *** *P* < 0.001.

## Data Availability

The datasets supporting the conclusions of this article are available in the TCGA and GEO cohorts.

## Abbreviations

RCC: Renal cell carcinoma
ccRCC: Clear cell renal cell carcinoma
VSX1: Visual system homeobox1
FKBP10: FK506-binding protein 10
TCGA: The cancer genome atlas
GEO: Gene expression omnibus
OS: Overall survival
DSS: Disease-specific survival
PFI: Progression-free interval
IHC: Immunohistochemistry
ACTB: β-Actin
KEGG: The Kyoto encyclopedia of genes and genomes
PRCC: Papillary renal cell carcinoma
