# Supplementary material for: High VSX1 expression promotes the aggressiveness of clear cell renal cell carcinoma by transcriptionally regulating FKBP10
Source: J Transl Med. 2022 Dec 3;20:554. doi: 10.1186/s12967-022-03772-2 (PMC9719260; doi:10.1186/s12967-022-03772-2)
Supplement: Supplementary file 1 — Additional file 1: Table S1. Primers sequence used for quantitative real-time PCR and shRNA sequence. [file 12967_2022_3772_MOESM1_ESM.docx]

**Table S1.** Primers sequence used for quantitative real-time PCR and shRNA sequence.

| Target  gene | | | Primer sequence (5’-3’) | | Size  (bp) |
| --- | --- | --- | --- | --- | --- |
|  |  |  | Forward | Reverse |  |
| VSX1 | | | GAGGCCCACTACCCTGATGT | TGTATCCGGTCTTCGGGGAG | 74 |
| TMEM44 | | | TTTCACTGGTGCCTACCTAGC | GGCTTCCCGATCTGAATTAGAC | 103 |
| FKBP10 | | | TACCACTACAACGGCACTTTTG | AGAACCACATCGAAGTAGAGGG | 93 |
| TRIB3 | | | AAGCGGTTGGAGTTGGATGAC | CACGATCTGGAGCAGTAGGTG | 127 |
| 18s rRNA | | | CAGCCACCCGAGATTGAGCA | TAGTAGCGACGGGCGGTGTG | 252 |
| Target  gene | | | shRNA sequence (5’-3’ Forward) | | |
| VSX1 | 1 | GCCAGGAAGTGAAGATAAGTT  GCTGCCAGACTCCGTGCTCAA  ACCACTTCAAAGAAGGTTCTA  CCACACCTACAATACCTATAT  GGACTTTGTTCGATACCATTA  CTACCACTACAACGGCACTTT | | | |
|  | 2 |  |  |  |  |
|  | 3 |  |  |  |  |
| FKBP10 | 1 |  |  |  |  |
|  | 2 |  |  |  |  |
|  | 3 |  |  |  |  |
