# Supplementary material for: High VSX1 expression promotes the aggressiveness of clear cell renal cell carcinoma by transcriptionally regulating FKBP10
Source: J Transl Med. 2022 Dec 3;20:554. doi: 10.1186/s12967-022-03772-2 (PMC9719260; doi:10.1186/s12967-022-03772-2)
Supplement: Supplementary file 3 — Additional file 3: Fig. S1. Verification of the impact of VSX1 on tumor cell invasiveness. (a−b) Tumor sphere formation assays evaluated the sphere-forming capacity for VSX1 overexpression or knockdown in 786-O and Caki-1 cells. (c−d) The Transwell assay evaluated cell migration and invasion of 786-O and Caki-1 cells with VSX1 overexpression or knockdown. Unpaired Student’s t-tests were used to assess the significance of differences. Data were presented as the mean ± standard deviation.*P < 0.05, ** P < 0.01, and *** P < 0.001. [file 12967_2022_3772_MOESM3_ESM.docx]

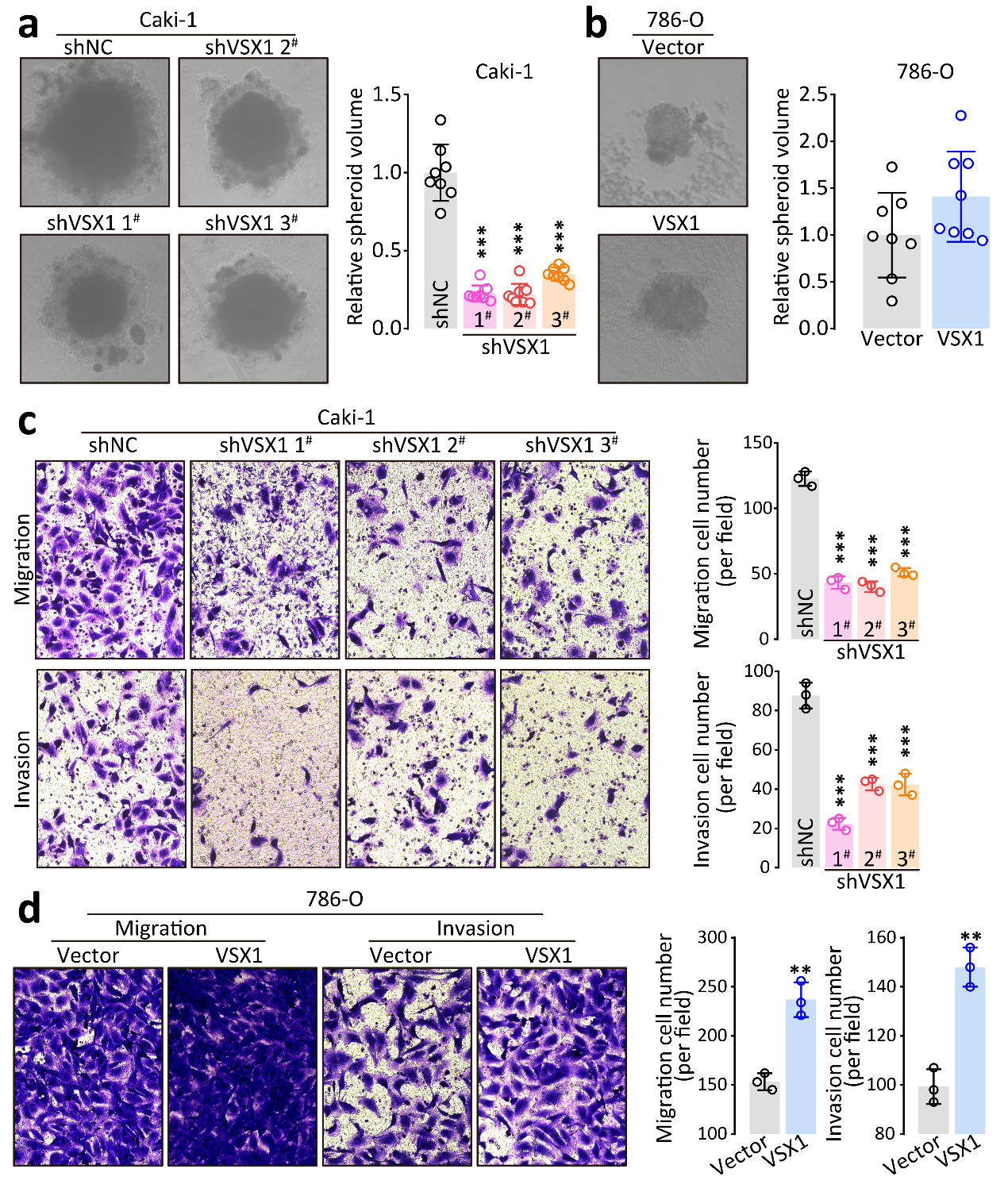


**Figure S1:** Verification of the impact of VSX1 on tumor cell invasiveness*.* (**a−b)** Tumor sphere formation assays evaluated the sphere-forming capacity for VSX1 overexpression or knockdown in 786-O and Caki-1 cells. **(c−d)** The Transwell assay evaluated cell migration and invasion of 786-O and Caki-1 cells with VSX1 overexpression or knockdown. Unpaired Student’s *t*-tests were used to assess the significance of differences. Data were presented as the mean ± standard deviation.**P* < 0.05, ** *P* < 0.01, and *** *P* < 0.001.
