# Supplementary material for: High VSX1 expression promotes the aggressiveness of clear cell renal cell carcinoma by transcriptionally regulating FKBP10
Source: J Transl Med. 2022 Dec 3;20:554. doi: 10.1186/s12967-022-03772-2 (PMC9719260; doi:10.1186/s12967-022-03772-2)
Supplement: Supplementary file 4 — Additional file 4: Fig. S2. Verification of the impact of VSX1 transcriptional activation on tumor cell invasiveness. (a) CCK-8 evaluated the proliferation of Caki-1 cells following FKBP10 knockdown. (b−c) The Transwell assay evaluated cell migration and invasion for FKBP10 knockdown in Caki-1 cells. (d) Representative colony formation of 786-O cells for FKBP10 knockdown after VSX1 overexpression. (e−f) The tumor sphere formation assay evaluated sphere-forming capacity of FKBP10 knockdown after VSX1 overexpression in 786-O cells. (g−h) The Transwell assay evaluated cell migration and invasion for FKBP10 knockdown after VSX1 overexpression in 786-O cells. Unpaired Student’s t-tests were used to assess the significance of differences. Data were presented as the mean ± standard deviation. *P < 0.05, ** P < 0.01, and *** P < 0.001. [file 12967_2022_3772_MOESM4_ESM.docx]

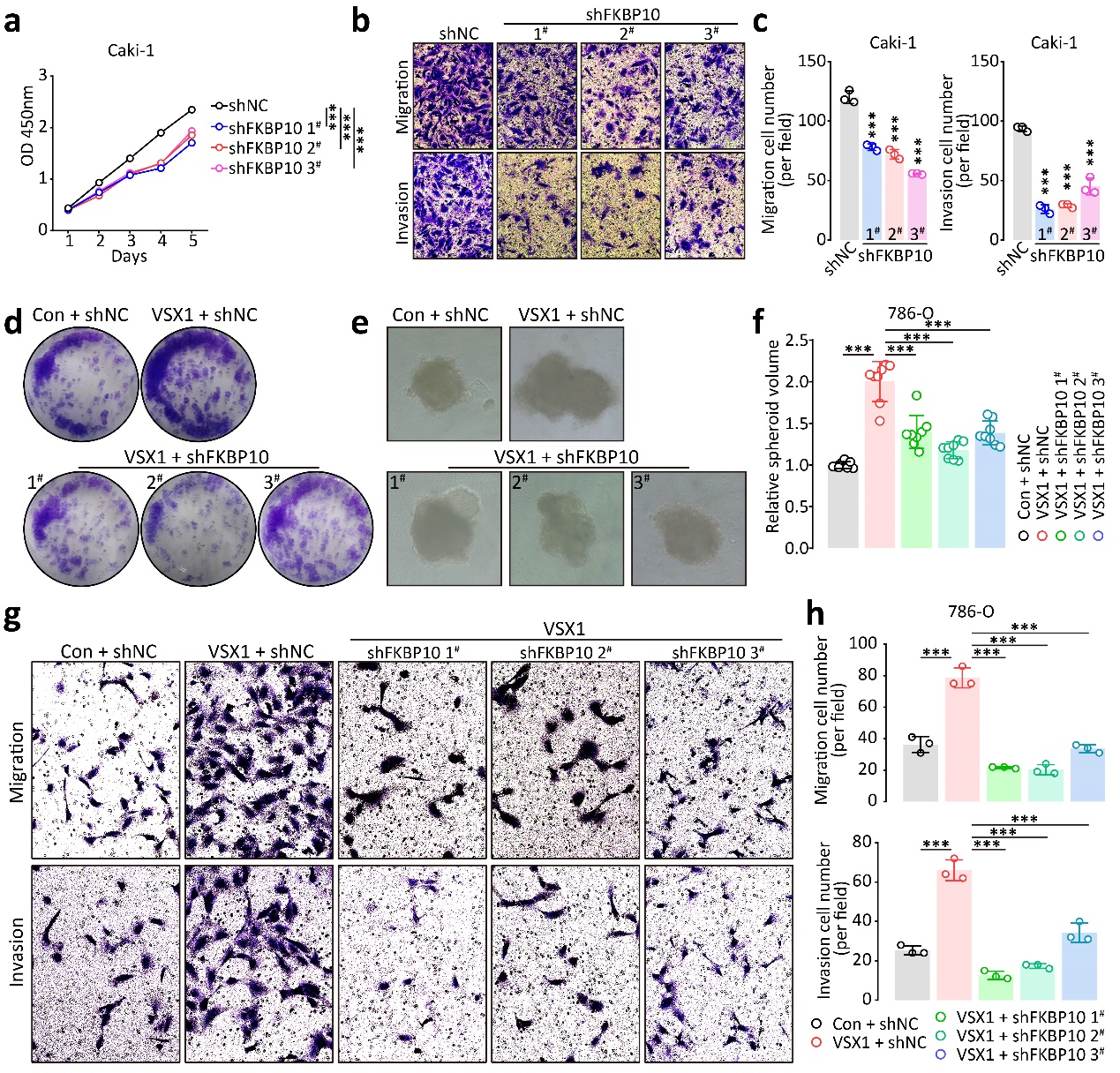


**Figure S2:** Verification of the impact of VSX1 transcriptional activation on tumor cell invasiveness*.* **(a)** CCK-8 evaluated the proliferation of Caki-1 cells following *FKBP10* knockdown. **(b−c)** The Transwell assay evaluated cell migration and invasion for *FKBP10* knockdown in Caki-1 cells. **(d)** Representative colony formation of 786-O cells for *FKBP10* knockdown after VSX1 overexpression. **(e−f)** The tumor sphere formation assay evaluated sphere-forming capacity of *FKBP10* knockdown after VSX1 overexpression in 786-O cells. **(g−h)** The Transwell assay evaluated cell migration and invasion for *FKBP10* knockdown after VSX1 overexpression in 786-O cells. Unpaired Student’s *t*-tests were used to assess the significance of differences. Data were presented as the mean ± standard deviation. **P* < 0.05, ** *P* < 0.01, and *** *P* < 0.001.
